# Prevalence of hyperglycaemia first detected during pregnancy and subsequent obstetric outcomes at St. Francis Hospital Nsambya

**DOI:** 10.1186/s13104-017-2493-0

**Published:** 2017-05-02

**Authors:** Betty Nakabuye, Silver Bahendeka, Romano Byaruhanga

**Affiliations:** 1 Department Obstetrics and Gynaecology, St. Francis Hospital Nsambya, P.O.Box 7146, Kampala, Uganda; 2 Mother Kevin Post Graduate Medical School, Uganda Martyrs University, P.O.Box 7146, Kampala, Uganda; 3 Department of Internal Medicine, Mother Kevin Post Graduate Medical School, Uganda Martyrs University, P.O.Box 7146, Kampala, Uganda

**Keywords:** Hyperglycaemia, Screening and pregnancy

## Abstract

**Background:**

Women with hyperglycaemia detected during pregnancy are at greater risk for adverse pregnancy outcomes. Data on hyperglycaemia in pregnancy in sub-Saharan Africa is scanty and varied depending on the populations studied and the methodologies used to define hyperglycaemia in pregnancy. With the recent 2013 World Health Organisation (WHO) diagnostic criteria and classification, there is yet no sufficient data on the prevalence of hyperglycaemia in sub-Saharan Africa. The objective was to determine the prevalence of Hyperglycaemia first detected during pregnancy and subsequent obstetric outcomes among patients attending antenatal care (ANC) at St. Francis Hospital Nsambya.

**Methods:**

A prospective cohort study. All women with no history of diabetes mellitus attending at or after 24 weeks gestation were eligible to participate in the study. Participants underwent a standard 75 g oral glucose tolerance test (OGTT) after an informed written consent. The primary outcome was diagnosis of hyperglycaemia. Enrolled participants were followed up to delivery to assess obstetric outcomes (secondary outcomes were birth weight, neonatal admission, maternal genital trauma, delivery mode, neonatal and maternal status at discharge).

**Results:**

251 women were screened between December 2013 and February 2014. The prevalence of hyperglycaemia first detected in pregnancy was 31.9%. We found 23.8 % of women with hyperglycaemia had no known risk factor. Macrosomia was the only obstetric outcome that was significantly associated with hyperglycaemia.

**Conclusion:**

The prevalence of hyperglycaemia first detected in pregnancy was high in the studied population. Clinicians, therefore, should become more vigilant to screen for the condition. Selective screening may miss 23.8% of pregnant women with hyperglycaemia. However the cost/benefit implications of screening strategy and the recent 2013 WHO diagnostic criteria need to be studied in our setting.

**Electronic supplementary material:**

The online version of this article (doi:10.1186/s13104-017-2493-0) contains supplementary material, which is available to authorized users.

## Background

During pregnancy, endocrine and metabolic changes occur that may predispose some women to hyperglycaemia, especially those whose pancreatic function cannot overcome these diabetogenic changes while pregnant [1].

Hyperglycaemia during pregnancy puts women at a higher risk of adverse outcomes like foetal macrosomia, obstructed labour, birth injuries, and maternal and perinatal mortality [2, 3]. Coupled with the above, is the long-term health impact of increased risk of developing type 2 diabetes. Cumulative risks of incident diabetes in gestational hyperglycaemic patients ranging from 2.6% to over 70% within 5–10 years of delivery have been reported [4–6]. Moreover, their off-springs have a higher prevalence of childhood obesity and overweight and higher risk of developing type 2 diabetes later in life [7, 8].

Over 371 million people have diabetes in the world and more than 14 million people in the African Region; by 2030 this is estimated to rise to 28 million [9]. Approximately half of these are women. Non-communicable diseases (NCDs) are on the rise in sub-Saharan Africa, but without well-established surveillance systems [10]. According to world health statistics, in Uganda, there is no country data available about factors associated with gestational hyperglycaemia. However, the estimate modelled using data from other countries and specific country characteristics showed the prevalence of raised fasting blood glucose among females aged ≥25 years as 6.5%; prevalence of raised blood pressure among women aged ≥25 years as 39.6% and women aged ≥20 years who are obese are about 4.9% [11]. This is predictive of gestational hyperglycaemia and its related events [12].

The prevalence of hyperglycaemia first detected in pregnancy varies worldwide and among ethnic groups depending upon the population studied and the used diagnostic tests. What is similar with the different studies is the fact that the prevalence has been increasing over time in women of different racial/ethnic backgrounds, possibly related to increases in mean maternal age and weight [13–20]. In 2013 the global prevalence of hyperglycaemia in pregnancy was estimated to be 16.9%, with 25.0% as the highest prevalence (South–East Asia) and lowest being 10.4% (North America and Caribbean Region). Low- and middle-income countries contribute 90% of the cases [21].

The diagnostic criteria for hyperglycaemia in pregnancy recommended by the World Health Organization in 1999 used non pregnant ranges with no evidence of their utility in pregnancy. WHO has therefore recently updated the diagnostic criteria and classification of hyperglycaemia first detected in pregnancy [22].

Therefore, hyperglycaemia first diagnosed at any time during pregnancy is currently classified [22] as diabetes mellitus in pregnancy or gestational diabetes. Diabetes in pregnancy is diagnosed if one’s fasting blood glucose ≥7.0 mmol/l and/or 2-h blood glucose ≥11.1 mmol/l following a 75 g oral glucose load while gestational diabetes mellitus is a fasting plasma glucose 5.1–6.9 mmol/l and/or 2-h plasma glucose 8.5–11.0 mmol/l following a 75 g oral glucose load.

The earlier definition included all levels of hyperglycaemia in one umbrella as Gestational diabetes being any degree of impaired glucose tolerance with onset or first recognition during pregnancy [22, 23].

In the 2006 WHO recommendations screening of gestational diabetes was between 24–28 weeks while in the 2013 recommendations screening is at any time during pregnancy [22, 23].

With both criteria, who to screen is still left to the attending health worker. Screening can be universal or selective. In selective screening, criteria utilized to identify those at increased risk of developing gestational hyperglycaemia includes: family history of diabetes, BMI > 30 kg/m^2^, delivery of a baby >4.0 kg, unexplained perinatal loss, and age > 35 years among others [24–28].

Information on hyperglycemia detected in pregnancy in Africa is limited [20, 22, 29]. Uganda lacks an organised screening programme for hyperglycemia in pregnancy though a few hospitals may offer selective screening for women with known risk factors a protocol that has been found to miss up to one-third of women with hyperglycaemia are at higher risk of adverse obstetric outcomes [30].

Because of the absence of a well organised screening programme, information on the prevalence of hyperglycemia in pregnancy and the obstetric outcomes of women diagnosed with the condition among women seeking antenatal care and delivery services at St. Francis Hospital Nsambya is lacking.

This study therefore determined the prevalence of hyperglycaemia first detected in pregnancy using the recent 2013 WHO diagnostic criteria and classification, and the obstetric outcomes among patients seeking obstetric care at St. Francis Hospital Nsambya since these have not been well established in our setting.

## Methods

Prospective cohort study conducted in the obstetrics and gynaecology department at St. Francis Hospital Nsambya. It is a faith based urban private not for profit hospital located about 3 km from the city centre in Kampala District, Uganda. The hospital offers specialist services in surgery, internal medicine, paediatrics, obstetrics and gynaecology.

The antenatal clinic (ANC) in the Obstetrics and Gynaecology department operates five days a week and on average has 500 new mothers monthly.

Total sample size was 251. The sample size was estimated using a standard formula on the basis of 10% prevalence of macrosomia based on recent estimates [31], 5% precision, 80% power and an expected response rate of 90% and including 10% loss to follow-up.

Included were pregnant women attending the antenatal clinic at St. Francis Hospital Nsambya at gestation age ≥ 24 weeks but ≤36 weeks calculated using menstrual dates or earliest ultrasound scan where menstrual dates were unknown, willing to give informed consent excluded known diabetic patients prior to the current pregnancy.

The study details were introduced to all mothers during the routine health talks at the antenatal clinic from 2nd December 2013 to 3rd February 2014. This was done on all days of the ANC. At registration desk, mothers whose pregnancies were estimated to be at 24 weeks of gestation or more were sent to a special room. In this room more written information about the study was given and gestation age was reassessed by the researcher. Gestation age was determined by first day of last normal menstrual period and/or obstetric ultrasound scan done by the hospital radiology team. Those who met the inclusion criteria were invited to participate after giving a written informed consent.

A standardised questionnaire was used to collect data on socio-demographic and risk factor profiles in the antenatal period and obstetrics outcomes at delivery and discharge (Additional file 1: Appendix 1). Data sources included information from the women, the women’s antenatal card, the patient delivery charts, the hospital delivery registers and their discharge notes.

Data on the following socio-demographic and risk factor variables was collected i.e. occupation, level of education, age, gravidity, parity, first degree relative with DM, gestational hypertension, pre-existing hypertension, history of first degree relative with high blood pressure, weight at first ANC, height, body mass index (BMI), gestation age at booking ANC visit, number of ANC visits before delivery, gestation age at screening for hyperglycemia, history of macrosomia, unexplained perinatal loss malformed baby, history of unexplained recurrent pregnancy loss, chronic drug use, history of failure to conceive, chronic illnesses.

Data on the primary outcome variable i.e. the prevalence of hyperglycemia first detected in pregnancy was collected after ascertaining the glycaemic status of all the enrolled women using the 75 g OGTT. The oral glucose tolerance test procedure and interpretation was done as follows:

1. The mothers were requested to have an overnight fast prior to the OGTT.
2. The site of blood sample collection (anterolateral aspect of the pulp of the left ring finger) was cleansed with 70% alcohol antiseptic and punctured. The initial blood flow was dried away with a dry piece of cotton.
3. Capillary blood samples were then collected.
4. The blood was analysed within 10 s using a glucose meter (Glucocard™ ∑-1070) for blood glucose concentration (check Additional file 2: Appendix 2: Specifications for the glucometer used (GlucocardTM ∑ GT-1070) for more specification of the glucose meter).
5. Fasting plasma glucose concentration was recorded in the questionnaire.
6. A 75 g oral glucose load flavoured with a quarter of an orange (to make it palatable, prevent nausea and vomiting) was given. The glucose solution was made by dissolving 75 g of anhydrous glucose in 300 ml of drinking water and a quarter of an orange. After drinking, mothers were requested to sit and rest for 2 h without ingesting any feed.
7. A 2 h plasma glucose concentration was then measured and recorded in the questionnaire.

### Interpretation of the 75 g OGTT test result [11]

1. A woman was normoglycaemic if both of the following criteria were met:
  Fasting blood glucose 5.1 mmol/l (<92 mg/dl) and
  2-h plasma glucose <8.5 mmol/l (<153 mg/dl) following a 75 g oral glucose load.
2. Diabetes in pregnancy was diagnosed if one or more of the following criteria were met:
  Fasting blood glucose ≥7.0 mmol/l (126 mg/dl).
  2-h blood glucose ≥11.1 mmol/l (200 mg/dl) following a 75 g oral glucose load.
3. Gestational diabetes mellitus was diagnosed if one or more of the following criteria were met:
  Fasting plasma glucose 5.1–6.9 mmol/l (92–125 mg/dl).
  2-h plasma glucose 8.5–11.0 mmol/l (153–199 mg/dl) following a 75 g oral glucose load.

The results were then given and explained to the patient and if hyperglycaemic, treatment was started depending on the glycaemic levels.

All mothers with hyperglycaemia were given advice on life style modification including 30–60 min walks daily, moderation of caloric intake and those with severe hyperglycaemia were started on insulin. Frequent monitoring with glucometers was encouraged and those who could afford bought them.

The routine antenatal care continued as usual.

The secondary outcomes were collected at delivery and discharge and included the mode of delivery, birth weight, genital tract trauma, and neonatal admission to nursery, perinatal and maternal status at discharge. The researcher reviewed all the maternity inpatient admissions, identified the charts of the women enrolled into the study, collected data on the gestational age at delivery, the mode of delivery, birth weight, genital tract trauma, and neonatal admission to nursery, perinatal and maternal status at discharge.

To minimise loss to follow up, the following strategies were employed to tracing the study participants;

- All the ANC cards of the participant were tagged with a label to indicate those being followed up in the study but also help the hospital staff identify them during care.
- Women were asked for the mobile phone contacts and encouraged to communicate with the researcher in form of a text message or a call during delivery especially if they did not deliver at the facility.
- All the participants were encouraged to deliver at the study site.
- A daily early morning check of all the maternity admissions and discharges was done by the researcher all through the duration of the study.

Permission from the hospital institutional review board and a written informed consent from the study participants were sought and obtained. Copies of the institutional review board approval letter and informed consent form are attached.

All the data was double entered into Epidata version 3.1, validated and cleaned. It was then exported to and analysed using SPSS software package version 19.0 (SPSS, Inc, Chicago, IL, USA). Data analysis was done for both numerical (quantitative) and categorical variables.

Continuous variables included the women’s age, gravidity, parity, gestation age, number of ANC visits, blood/plasma glucose concentration and babies’ birth weight.

The numerical variables were explored in SPSS with descriptive statistics and assessed for normality using histograms with normal curves overlying. They were also summarised using means and standard deviations. Further analysis was done to compare mean between normal glycaemia and hyperglycaemic mothers. Data was then categorised into: Age groups (≤24, 25–29, 30–34 and ≥35 years), BMI groups {(<25 and ≥25 kg/m^2^) and ≤30 and >30 kg/m^2^}.

Discrete numerical data analysed included gravidity, parity and number of ANC visits. These were explored using mean, median, and mode and quartiles. They were further categorised into: Gravidity {prime-gravida and multigravida (≥2 pregnancies)}, parity {≤1 (prime-para), 2–4 (multipara) and ≥5 (grand-multipara)}, number of ANC visits (<4 and ≥4), and birth weight (4.0 and >4.0 kg).

Categorical data was summarised using frequency tables and percentages. The relationship between variables and outcomes were explored using 2 × 2 contingency tables to determine Chi squares and associated p-values. Further analysis was done using logistic regression and multivariate logistic regression analysis.

## Results

The new ANC attendance during the study period was 1201, and 633 were potentially eligible mothers in that time period but 401 were assessed for eligibility, 333 were confirmed eligible and 251 consented for the test. Those who were never willing to do the test were mothers who did not have enough time sit through the two hours of the OGTT. Therefore, the response rate was 75.4% (no information on these women is available). Twenty-seven (10.8%) of those who were tested were lost to follow up.

**Fig. 1:**
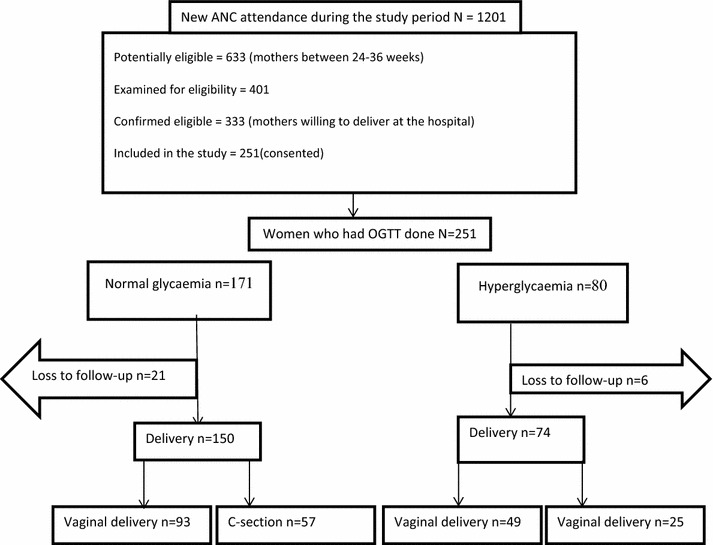
Study profile

These either did not deliver from the study site or their delivery details could not be retrieved from the hospital records (Fig. 1).

A total of 251 pregnant women of gestation age 24–36 weeks were enrolled in the study and all completed the OGTT. Their demographic and obstetric characteristics are summarised in Table 1. All the continuous variables like age, BMI showed normal distribution.

**Table 1:** Summary of baseline demographic data (N = 251)

Maternal variable	
Age in years mean (sd)	29.25 (4.97)
BMI (kg/m^2^) mean (sd)	26.81 (4.63)
Gestation age at booking visit mean (sd)	23.36 (6.70)
Gestation age at OGTT screening mean (sd)	32.64 (2.89)
Gravidity n (%)	
Primegravida	50 (19.9)
Parity n (%)	
≤1	130 (51.8)
2–4	108 (43.0)
≥5	13 (5.2)
Age groups (years) n (%)	
≥35	44 (17.5)
BMI (kg/m^2^) n (%)	
≥25	153 (61.0)
Occupation n (%)	
Unskilled	87 (34.7)
Skilled	98 (39.0)
Unemployed	66 (26.3)
Level of education n (%)	
Primary level and below	31 (12.4)
Secondary level	87 (34.7)
Tertiary	133 (53.0)
HIV positive n = 249	12 (4.8)
Number of ANC visits n = 224 (%)	
<4	85 (33.9)
≥4	139 (55.4)
Presence of risk factors to hyperglycaemia	
No risk factor	91 (36.3)
≥1 risk factor	160 (63.7)

The mean (sd) age of participants was 29.25 (4.97) years with 82% being less than 35 years. 39% of all mothers were doing skilled jobs, 34% unskilled and 26.3% were unemployed. One hundred thirty-three (53%) received tertiary education. Fifty (19.9%) of the enrolled mothers were carrying their first pregnancy. The mean (sd) gestation ages at booking visit and OGTT screening were 23.36 (6.71) and 32.6 (2.91) weeks respectively. The mean (sd) BMI at first ANC visit was 26.8 (4.63) kg/m^2^ with 61% of them being at least overweight (BMI ≥ 25 kg/m^2^). Sixty-four (25.5%) of the studied women had positive family history of diabetes in a first degree relative, and 18.3% did not know if they had this history. Twelve participants (4.8%) were HIV positive and no participant had a positive result for TPHA, 139 (55.4%) had at least 4 ANC visits before delivery and 160 (63.7%) of the participants had at least one risk factor associated with hyperglycaemia (Table 1).

### Prevalence of hyperglycaemia

All participants who had the OGTT done completed the test and their results are summarised in Table 2. The prevalence of hyperglycaemia first detected in pregnancy was 31.9 with 95% of the hyperglycaemic women classified as having gestational diabetes (GDM) and only 5% with diabetes first diagnosed in pregnancy. The mean (sd) fasting plasma glucose was 4.7 (0.8) mmol/l and the mean (sd) 2-h plasma glucose was 6.9 (1.5) mmol/l.

**Table 2:** Oral glucose tolerance test (OGTT) results N = 251

	Mean (sd)
Fasting plasma glucose (mmol/l)	4.71 (0.82)
2-h plasma glucose concentration (mmol/l)	6.88 (1.51)

	n (%)
Normal glycaemia	171 (68.1)
Hyperglycaemia	80 (31.9)
GDM	76 (95)
DM in pregnancy	4 (5)

The mean (sd) fasting plasma glucose for those diagnosed with hyperglycaemia was 8.1 (1.8) mmol/l. Fifteen (6%) of the participants reported nausea but none vomited. Only one patient reported palpitations. All patients diagnosed with hyperglycaemia were treated with diet and exercise. Only 5% of the hyperglycaemic patients received insulin and these were the same women who had diabetes in pregnancy. Although the two (GDM and diabetes in pregnancy) are reported, for the main analyses below all hyperglycaemia is presented.

### **R**isk factors associated with hyperglycaemia

Univariate logistic regression analysis revealed statistically significant differences in parity, BMI at first ANC visit and family history of hypertension between both groups (normoglycaemic and hyperglycaemic mothers).

The presence of at least one risk factor to hyperglycaemia was strongly associated with the diagnosis p value = 0.005; odds ratio 2.34 (95% CI 1.28–4.25) (Table 3). However 19 (23.8%) of women with hyperglycaemia had no known risk factors. Risk factors assessed were age, family history of diabetes, booking body mass index over 30 kg/m^2^, age of 35 years and above, history of macrosomia, previous unexplained perinatal loss, birth of a malformed child, failure to conceive, previous history of gestational hyperglycaemia, essential hypertension and pregnancy-related hypertension (some not presented in the table because they were not significant).

**Table 3:** Summary of the results of Univariate logistic regression analysis of risk factors associated with hyperglycaemia N = 251

	Normoglycaemia	Hyperglycaemia	Chi square	Df	p-value	OR (95% CI)
Age in years: mean (sd)	29.08 (4.88)	29.61 (5.17)			0.43	
Gestation age at OGTT (weeks) mean (sd)	32.64 (2.78)	32.62 (3.14)			0.74	
BMI: mean (sd)	26.31 (4.41)	27.88 (4.96)			0.01	
Presence of at least one risk factor n (%)			8.26	1	0.005	
No	72 (42.1)	19 (23.8)				Ref
Yes	99 (57.9)	61 (76.3)				2.34 (1.28–4.25)
Parity n (%)			8.19	2	0.03	
≤1	90 (52.6)	40 (50.0)				Ref
2–4	77 (45.0)	31 (38.8)				0.91 (0.52–1.58)
≥5	4 (2.3)	9 (11.3)				5.06 (1.47–17.41)
Age groups (years) n (%)			1.02	3	0.80	
<25	35 (20.5)	16 (20.0)				Ref
25–29	60 (35.1)	24 (30.0)				0.88 (0.41–1.87)
30–34	46 (26.9)	26 (32.5)				1.24 (0.58–2.65)
≥35	30 (17.5)					1.02 (0.41–2.43)
BMI: n (%)			5.10	1	0.02	
≤30	138 (80.7)	54 (67.5)				Ref
>30	33 (19.3)	26 (32.5)				2.01 (1.10–3.68)
Family history of DM n (%)			2.27	2	0.01	
No	106 (62.0)	35 (43.8)				Ref
Yes	35 (20.5)	29 (36.3)				2.51 (1.35–4.68)
Unknown	30 (17.5)	16 (20.0)				1.62 (0.79–3.31)
History of macrosomic baby (>4 kg) n (%)			0.88	1	0.34	
No	145 (84.8)	64 (80.0)				Ref
Yes	26 (15.2)	16 (20.0)				1.39 (0.70–2.78)

Women with BMI > 30 kg/m^2^ were 2.01 times (95% CI 1.1–3.68) more likely to have hyperglycaemia compared to those with BMI ≤ 30 kg/m^2^ and this difference was statistically significant p-value = 0.02. Those with a positive family history of (first degree relative with) DM were 2.51 times (95% CI 1.35–4.68) more likely compared to those who had none 16(34.8%) of the hyperglycaemic women were not sure of DM family history. These women were 1.62 times (95% CI 0.79–3.31) more likely to have hyperglycaemia compared to those who reported no history. Grand-multiparous women were 5.06 times more at risk of being diagnosed with gestational hyperglycaemia compared with those with parity ≤1, p-value = 0.03, (95% CI 1.47–17.41). Maternal age, family history of hypertension, history of macrosomic baby perinatal death and gestation age at OGTT were not associated with hyperglycaemia. 19 (20.9%) of mothers diagnosed with hyperglycaemia had no known risk factors to hyperglycaemia. The presence of at least one risk factor to hyperglycaemia was strongly associated with the diagnosis p-value = 0.005; odds ratio 2.34 (95% CI 1.28–4.25) (Table 3).

However at multivariate analysis, none of risk factors (parity, BMI and family history of DM) was significantly associated with hyperglycaemia (Table 4).

**Table 4:** Multivariate analysis of the risk factors associated with hyperglycaemia first detected in pregnancy

Maternal variable	Wald Chi square	Df	p-value	OR (95% CI)
Parity	4.69	2	0.10	
≤1				Ref
2–4			0.87	0.32 (0.09–1.18)
≥5			0.03	0.25 (0.07–0.90)
BMI	2.72	1	0.10	
≤30				Ref
>30				0.58 (0.30–1.10)
Family history of DM	5.91	2	0.05	
No				Ref
Yes			0.34	0.70 (0.33–1.47)
Unknown			0.29	1.54 (0.69–3.46)

### Obstetric outcomes

Of the 251 screened mothers, 224 (89.2%) delivered from the study site and their delivery details were available. Therefore the data presented in Table 5 is for only those whose records were available at the hospital. Genital tract trauma was assessed in only those who had vaginal delivery. This is why the number assessed was 134. This number is less than the total vaginal deliveries because some data was missing on whether one had trauma or not. Two babies were not weighed at birth and their birth weight was therefore not known. This is why there are 222 babies assessed for macrosomia. Five babies were intra-uterine deaths and therefore were not assessed for neonatal admission because they did not have a chance at admission. Macrosomia was significantly associated with hyperglycaemia p-value = 0.003.

**Table 5:** Summary of maternal and perinatal outcomes (gestational hyperglycaemia vs. normal) N = 224

Variable	Normal OGTT	Hyperglycaemia	p-value
Gestation age at delivery (weeks): mean (sd)	39.19 (1.57)	39.09 (2.08)	0.10
Mode of delivery: n (%)			0.54
Vaginal delivery	93 (62.0)	49 (66.2)	
C-section	57 (38.0)	25 (34.8)	
Genital tract trauma n = 134			0.78
Yes (n %)	47 (50.5)	26 (53.1)	
No (n %)	46 (49.5)	23 (46.9)	
Birth weight (kg) mean (sd)	3.25 (0.44)	3.30 (0.60)	0.03
Birth weight (kg) n = 222			0.003
≤4.0	147 (99.3)	68 (91.9)	
>4.0	1 (0.7)	6 (8.1)	
Neonatal admission n = 219			0.60
Yes n (%)	38 (25.9)	21 (29.2)	
No n (%)	109 (74.1)	51 (70.8)	
Status of baby at discharge n (%)			0.37
Alive	147 (98.0)	71 (95.9)	
Dead	3 (2.0)	3 (4.1)	

Although the hyperglycaemic group had higher percentages of caesarean section, neonatal admission and genital tract trauma, these differences were too small to have a statistical significance (Table 5). There was no maternal death in this cohort.

## Discussion

The prevalence of hyperglycaemia first detected in pregnancy in this study was 31.9%. Hyperglycaemic mothers were at a higher risk delivering macrosomic babies compared to normal glycaemic ones. A significant number 23.8% of mothers with hyperglycaemia had no known risk factor to hyperglycaemia.

This study looked at all pregnant women attending ANC without selecting those with risk factors. We also the assessed presence of a number of risk factors age, family history of diabetes, booking body mass index over 30 kg/m^2^, age of 35 years and above, history of macrosomia, previous unexplained perinatal loss, birth of a malformed child, failure to conceive, previous history of gestational hyperglycaemia, essential hypertension and pregnancy-related hypertension. It identified women with both GDM and diabetes in pregnancy separately, in accordance with the latest WHO guidance.

The prevalence of hyperglycaemia reported in this study is higher than that reported in other studies [32, 33]. The worldwide prevalence hyperglycaemia in pregnancy was estimated to be about 15% [21]. However WHO has estimated that with its recent diagnostic criteria the prevalence of hyperglycaemia is expected to be higher because of the lowered thresholds for diagnosis [22]. A study done in Tanzania [32] a prevalence of hyperglycaemia found to be 0%. It, however, used higher thresh hold for diagnosis compared to the criteria used in this study. Secondly it was done over 20 years ago and yet hyperglycaemia is increasing over time [22]. The mean fasting and 2 h plasma glucose levels from this study are higher than that reported among the Tanzanian women (4.7 versus 3.5 mmol/l and 6.9 versus 4.2 mmol/l) [32]. Another study in rural Ethiopia found a prevalence 3.9% [33]. It also had a higher threshold for diagnosis and was done over 10 years ago. Interestingly the mean 2 h plasma glucose (for those diagnosed with hyperglycaemia) for both studies were not markedly different (8.6 mmol/l in Ethiopia versus 8.1 mmol/l in Uganda) indicating that it was probably a difference in the diagnostic criteria that is responsible for the observed differences. Secondly both the Tanzanian and Ethiopian studies were done in rural women unlike this study which was done among urban dwellers of Uganda. This may also explain the noted differences.

A study done in developed countries found prevalence rates of gestational hyperglycaemia ranging from less than 1 to 20% [34]. It also found significant differences in screening and diagnostic approaches among countries affecting the reported differences in prevalence. In Africa, Hall et al. [20] in a systematic review noted scanty data on hyperglycaemia. It found the prevalence of gestation hyperglycaemia ranging from 0% in Tanzania to 9% in Ethiopia. These studies had different diagnostic criteria as well.

The screening strategy used in this study was universal screening where screening was done for every pregnant woman presenting at the ANC after 24 weeks of gestation including those with no known risk factors. Selective screening strategy using risk factor profile misses up to 45% of mothers with gestational hyperglycaemia [30, 35–37]. This may also be another explanation for this high prevalence since universal screening (without using risk factors) was used in this study.

In this study, 19 (23.8%) of participants with hyperglycaemia had no known risk factor associated with gestational hyperglycaemia, just like in a study in France where it was noted that a selective screening would lead to missing one-third of the women with GDM who, even without risk factors, had more GDM related events [30].

In this study, however, hyperglycaemia was not strongly associated with these poor obstetric outcomes apart from macrosomia. This may have been because the frequencies of these outcomes were so few that they could not give a statistical significance [22, 38–41].

### Generalizability

These results may only apply to the studied population, that is, to pregnant women of 24 or more weeks of gestation. It was done in a private not for profit hospital. This study was a hospital based study and the participants were voluntarily tested. The results presented here may not apply to population based studies where participants’ selection is random.

## Limitations

Capillary blood samples were used because the study could not meet the cost of testing glucose concentrations on venous blood samples. Glucose meters have limitations in the diagnosis of hyperglycaemia. The accuracy of glucose meter results is dependant operator technique, environmental exposure, and patient physiologic and medication effects. For this study, blood testing was done by the researcher ensuring that the stated procedure by the manufacturer was followed. The glucose meters and the test strips were kept in their container that prevented them from environmental hazards. Our study participants were stable out-patients whose physiology was not expected to alter reading. None had medication, oxygen therapy, anaemia, hypotension that would affect the result.

With-holding treatment for hyperglycaemic study participants could not be done for ethical reasons yet treatment improves obstetric outcomes that were assessed. This therefore, may have affected the reported results about the obstetric outcomes.

This study was a hospital based study and the participants were voluntarily tested. The results presented here may not apply to population based studies where participants’ selection is random.

## Conclusion

The prevalence of hyperglycaemia first detected in pregnancy was 31.9 with 95% of them being diagnosed with GDM and 5% DM in pregnancy. Selective screening of women with only risk factors may miss up to 23.8% women with the condition in our setting. Despite treatment, women with hyperglycaemia still gave birth to bigger babies. Clinicians should be more vigilant to screen mothers with hyperglycaemia. The hospital should consider universal screening of hyperglycaemia first detected in pregnancy. However cost/benefit implications of screening strategies and the recent 2013, WHO diagnostic criteria and classification of hyperglycaemia first detected in pregnancy need to be studied in our setting.

## Additional files

**Additional file 1: Appendix 1.** Questionnaire.

**Additional file 2: Appendix 2.** Specifications for the glucometer used (GlucocardTM ∑ GT-1070).

## Abbreviations

ANC: antenatal clinic
CDC: Centre for Disease Control
BMI: body mass index
DM: diabetes mellitus
GDM: gestational diabetes mellitus
OGTT: oral glucose tolerance test
NCD: non-communicable diseases
TPHA: *Treponema palladium* haemo-agglutination test
WHO: World Health Organisation

## References

[1] Butte NF (2000). Carbohydrate and lipid metabolism in pregnancy: normal compared with gestational diabetes mellitus. Am J Clin Nutr.

[2] Dodd JM, Crowther CA, Antoniou G, Baghurst P, Robinson JS (2007). Screening for gestational diabetes: the effect of varying blood glucose definitions in the prediction of adverse maternal and infant health outcomes. Aust NZ J Obstet Gynaecol.

[3] Schmidt MI, Duncan BB, Reichelt AJ, Branchtein L, Matos MC, e Forti AC (2001). Gestational diabetes mellitus diagnosed with a 2-h 75-g oral glucose tolerance test and adverse pregnancy outcomes. Diabetes Care.

[4] Chodick G, Elchalal U, Sella T, Heymann A, Porath A, Kokia E (2010). The risk of overt diabetes mellitus among women with gestational diabetes: a population-based study. Diabet Med.

[5] Kim C, Newton KM, Knopp RH (2002). Gestational diabetes and the incidence of type 2 diabetes a systematic review. Diabetes Care.

[6] Kaaja RJ, Greer IA (2005). Manifestations of chronic disease during pregnancy. JAMA.

[7] Dabelea D (2007). The predisposition to obesity and diabetes in offspring of diabetic mothers. Diabetes Care.

[8] Ornoy A (2005). Growth and neurodevelopmental outcome of children born to mothers with pregestational and gestational diabetes. Pediatr Endocrinol Rev: PER..

[9] IDF (2012). Diabetes atlas.

[10] Abegunde DO, Mathers CD, Adam T, Ortegon M, Strong K (2007). The burden and costs of chronic diseases in low-income and middle-income countries. Lancet.

[11] World Health Organization. World health statistics. http://www.who.int/gho/publications/world_health_statistics/2013/en/. Accessed 2013.

[12] Hedderson MM, Ferrara A (2008). High blood pressure before and during early pregnancy is associated with an increased risk of gestational diabetes mellitus. Diabetes Care.

[13] O’Sullivan JB, Mahan CM (1964). Criteria for the oral glucose tolerance test in pregnancy. Diabetes.

[14] Amankwah KS, Prentice RL, Fleury FJ (1977). The incidence of gestational diabetes. Obstet Gynecol.

[15] Mestman JH (1980). Outcome of diabetes screening in pregnancy and perinatal morbidity in infants of mothers with mild impairment in glucose tolerance. Diabetes Care.

[16] Carpenter MW, Coustan DR (1982). Criteria for screening tests for gestational diabetes. Am J Obstet Gynecol.

[17] Hadden D (1985). Geographic, ethnic, and racial variations in the incidence of gestational diabetes mellitus. Diabetes.

[18] Getahun D, Nath C, Ananth CV, Chavez MR, Smulian JC (2008). Gestational diabetes in the United States: temporal trends 1989 through 2004. Am J Obstet Gynecol..

[19] Dabelea D, Snell-Bergeon JK, Hartsfield CL, Bischoff KJ, Hamman RF, McDuffie RS (2005). Increasing prevalence of gestational diabetes mellitus (GDM) over time and by birth cohort Kaiser permanente of Colorado GDM screening program. Diabetes Care.

[20] Hall V, Thomsen RW, Henriksen O, Lohse N (2011). Diabetes in sub Saharan Africa 1999-2011: epidemiology and public health implications. A systematic review. BMC Public Health.

[21] Guariguata L, Linnenkamp U, Beagley J, Whiting D, Cho N (2014). Global estimates of the prevalence of hyperglycaemia in pregnancy. Diabetes Res Clin Pract.

[22] WHO (2013). Diagnostic criteria and classification of hyperglycaemia first detected in pregnancy.

[23] WHO (2006). Definition and diagnosis of diabetes mellitus and intermediate hyperglycemia: report of a WHO/IDF consultation.

[24] Solomon CG, Willett WC, Carey VJ, Rich-Edwards J, Hunter DJ, Colditz GA (1997). A prospective study of pregravid determinants of gestational diabetes mellitus. JAMA.

[25] Saldana TM, Siega-Riz AM, Adair LS, Suchindran C (2006). The relationship between pregnancy weight gain and glucose tolerance status among black and white women in central North Carolina. Am J Obstet Gynecol.

[26] Hedderson MM, Williams MA, Holt VL, Weiss NS, Ferrara A (2008). Body mass index and weight gain prior to pregnancy and risk of gestational diabetes mellitus. Am J Obstet Gynecol.

[27] Herring SJ, Oken E, Rifas-Shiman SL, Rich-Edwards JW, Stuebe AM, Kleinman KP (2009). Weight gain in pregnancy and risk of maternal hyperglycemia. American journal of obstetrics and gynecology..

[28] Tovar A, Must A, Bermudez OI, Hyatt RR, Chasan-Taber L (2009). The impact of gestational weight gain and diet on abnormal glucose tolerance during pregnancy in Hispanic women. Matern Child Health J.

[29] Odar E, Wandabwa J, Kiondo P (2004). Maternal and fetal outcome of gestational diabetes mellitus in Mulago Hospital, Uganda. Afr Health Sci.

[30] Cosson E, Benbara A, Pharisien I, Nguyen MT, Revaux A, Lormeau B (2013). Diagnostic and prognostic performances over 9 years of a selective screening strategy for gestational diabetes mellitus in a cohort of 18,775 subjects. Diabetes Care.

[31] Langer O, Berkus MD, Huff RW, Samueloff A (1991). Shoulder dystocia: should the fetus weighing ≥ 4000 grams be delivered by cesarean section?. Am J Obstet Gynecol.

[32] Swai A, Kitange H, McLarty D, Kilima P, Masuki G, Mtinangi B (1991). No deterioration of oral glucose tolerance during pregnancy in rural Tanzania. Diabet Med.

[33] Seyoum B, Kiros K, Haileselase T, Leole A (1999). Prevalence of gestational diabetes mellitus in rural pregnant mothers in northern Ethiopia. Diabetes Res Clin Pract.

[34] Jiwani A, Marseille E, Lohse N, Damm P, Hod M, Kahn JG (2012). Gestational diabetes mellitus: results from a survey of country prevalence and practices. J Matern-Fetal Neonat Med..

[35] Östlund I, Hanson U (2003). Occurrence of gestational diabetes mellitus and the value of different screening indicators for the oral glucose tolerance test. Acta Obstet Gynecol Scand.

[36] Hiéronimus S, Le Meaux J-P (2010). Relevance of gestational diabetes mellitus screening and comparison of selective with universal strategies. Diabetes Metab..

[37] Beucher G, Viaris de Lesegno B, Dreyfus M (2010). Maternal outcome of gestational diabetes mellitus. Diabetes Metab..

[38] Sullivan J, Gellis S, Dandrow R, Tenney B (2003). The potential diabetic and her treatment in pregnancy. Obstet Gynecol..

[39] Crowther CA, Hiller JE, Moss JR, McPhee AJ, Jeffries WS, Robinson JS (2005). Effect of treatment of gestational diabetes mellitus on pregnancy outcomes. N Engl J Med.

[40] Bonomo M, Corica D, Mion E, Goncalves D, Motta G, Merati R (2005). Evaluating the therapeutic approach in pregnancies complicated by borderline glucose intolerance: a randomized clinical trial. Diabet Med.

[41] Langer O, Yogev Y, Most O, Xenakis EM (2005). Gestational diabetes: the consequences of not treating. Am J Obstet Gynecol.

