# Supplementary material for: Prevalence of hyperglycaemia first detected during pregnancy and subsequent obstetric outcomes at St. Francis Hospital Nsambya
Source: BMC Res Notes. 2017 May 2;10:174. doi: 10.1186/s13104-017-2493-0 (PMC5414152; doi:10.1186/s13104-017-2493-0)
Supplement: Supplementary file 1 — Additional file 1: Appendix 1. Questionnaire. [file 13104_2017_2493_MOESM1_ESM.docx]

**Appendix 1: QUESTIONNAIRE**

Thank you for accepting to be part of this study.

It is to find out the magnitude of mothers with high blood sugar in pregnancy its effects on these mothers. All the information given will be kept confidential and only used for this research purposes. Your participation will not affect the care you receive from here.

**SECTION A: IDENTIFICATION**

1. Study number
2. IP
3. Address
4. Telephone contact
5. ANC booking: 1. General 2. Private

**SECTION B: SOCIAL DEMOGRAPHIC AND FAMILY CHARACTERISTICS**

1. Occupation
2. Level of education: 1. None 2. Primary 3. Secondary 4. Tertiary
3. Age
4. Gravida
5. Parity
6. Do you have diabetes Mellitus? 1. Yes 2. No 3. Do not know
7. First degree relative with high blood sugar 1. Yes 2. No 3. Do not know
8. History of high blood pressure during pregnancy 1. Yes 2. No
9. History of high blood pressure during this pregnancy 1. Yes 2. No
10. History of high blood pressure while not pregnant 1. Yes 2. No
11. History of first degree relative with high blood pressure 1. Yes 2. No 3. Do not know
12. Weight at first ANC
13. Height
14. BMI
15. Gestation age at first ANC
16. Number of ANC before delivery
17. Gestation age at OGTT
18. IPT (malaria doses) 1. One dose 2. Two doses 3. None
19. TPHA: 1. Reactive 2. Non-reactive 3. Not done
20. HIV serology: 1. Positive 2. Negative 3. Not done

**OBSTETRIC HISTORY**

1. Any baby weighing more than 4kg? 1. Yes 2. No
2. How many babies weighing 4kg and above?
3. Unexplained perinatal loss 1. Yes 2. No
4. Malformed baby 1. Yes 2. No
5. History of unexplained recurrent pregnancy loss 1. Yes 2. No
6. Chronic Drugs
7. History of failure to conceive 1. Yes 2. No
8. State any chronic illnesses that you have

SECTION C: OGTT

1. Fasting plasma glucose
2. 2 hour plasma glucose
3. Gestational hyperglycaemia 1. Yes 2. No
4. What type of gestational hyperglycaemia 1. GDM 2. DM
5. Complication of OGTT 1. Yes 2. No
6. If yes specify

SECTION D: MANAGEMENT OF GESTATIONAL HYPERGLYCAEMIA

1. Treatment given
2. Life style modification (diet and exercise)
3. Insulin
4. 1 and 2
5. Others

SECTION E: DELIVERY DETAILS

1. Gestational age at delivery
2. Type of delivery:

1. Normal vaginal delivery

2. Vacuum extraction

3. Breech delivery

3. Caesarean section

5. Others (specify)

1. Birth weight
2. Is birth weight >4.0kg 1. Yes 2. No
3. Genital tract trauma 1. Yes 2. No
4. Admission to Nursery 1. Yes 2. No
5. Perinatal Death 1. Yes 2. No
6. If yes in 42 above, specify below
7. MSB 2. FSB 3. Early Neonatal death
8. Outcome of mother 1. Alive 2. Dead
