# Supplementary material for: Prevalence of hyperglycaemia first detected during pregnancy and subsequent obstetric outcomes at St. Francis Hospital Nsambya
Source: BMC Res Notes. 2017 May 2;10:174. doi: 10.1186/s13104-017-2493-0 (PMC5414152; doi:10.1186/s13104-017-2493-0)
Supplement: Supplementary file 2 — Additional file 2: Appendix 2. Specifications for the glucometer used (GlucocardTM ∑ GT-1070). [file 13104_2017_2493_MOESM2_ESM.docx]

## Appendix 2: Specifications for the glucometer used (Glucocard^TM^ ∑ GT-1070)

| Product | Glucocard^TM^ ∑ |
| --- | --- |
| Model | GT-1070 |
| Test Item | Blood glucose level |
| Sample | Fresh capillary blood(although whole blood samples are used for measurement, displayed results are equivalent to plasma glucose levels) |
| Sample size | 0.5µL |
| Test strips | Glucocard **^TM^ ∑**test strips |
| Unit of measure | mmol/L |
| Test range | 0.6 to 33.3 mmol/L |
| Test time | 7 seconds from blood detection |
| Temperature compensation | Automatic compensation using built-in thermo sensor |
| Operating environment | Temperature: 10 to 40^0^C  Humidity: 20 to 80%(no condensation) |
